# Systematic Review of Pregnancy After Myomectomy: The Risk of Uterine Rupture

**DOI:** 10.1155/ogi/6048559

**Published:** 2026-10-17

**Authors:** Camille Groffils, Lena Paelinck, Steffi van Wessel, Jan Bosteels, Kristien Roelens, Griet Vandenberghe

**Affiliations:** ^1^ Department of Obstetrics and Gynecology Ghent University Hospital Ghent Belgium uzgent.be

## Abstract

**Introduction:**

A myoma is the most common pelvic mass in the urogenital tract and can cause various symptoms. Myomectomy is sometimes performed as treatment. A myomectomy prior to pregnancy can cause some risks or complications. The most feared complication is a uterine rupture, with an incidence of 0.60%–0.75% reported in the literature up to 2012. The aim of this study is to review recent literature on the risk of uterine rupture during labour following a myomectomy.

**Materials and Methods:**

To identify new insights, an extensive literature search was performed in the following databases: MEDLINE (PubMed), Embase, Web of Science and Cochrane Library. Articles published between 1 April 2013 and 31 October 2023 were included. The articles were screened for inclusion or exclusion by two reviewers. A total of 31 articles were included.

**Results:**

A total of 13,381 pregnant women with a history of myomectomy were included. The estimated overall risk of uterine rupture during pregnancy and labour was 0.30% (40/13.381; 95% CI: 0.21–0.41). Th included studies did not provide sufficient data to determine the specific risk of uterine rupture during a trial of labour after myomectomy (TOLAM). The risk of uterine rupture was significantly higher (*p* = 0.04) when the uterine cavity had been entered during the myomectomy (3/44 or 6.82%) compared to when it was not entered (4/336 or 1.19%) with an odds ratio of 6.07. The type of myomectomy (laparoscopic, open or robotic) did not significantly influence the risk. No consistent data were available to identify other potential risk factors.

**Conclusion:**

This systematic review found an incidence of uterine rupture during pregnancy and labour of 0.30% after a previous myomectomy. Entry into the uterine cavity during myomectomy appears to significantly increase the risk. Large, high‐quality prospective studies are needed to confirm this finding and to identify additional risk factors.

## 1. Introduction

Uterine myomas are the most common benign pelvic tumours in women of reproductive age, with a prevalence of 20%–25% in this group overall and up to 30%–40% in women over 40 years of age [1–3].

The main risk factors for myomas are age, black race and a family history of myomas [2, 3].

Myomas originate from smooth muscle cells and fibroblasts of the myometrium. Myomas can vary in size, weight, number and location.

The International Federation of Gynaecology and Obstetrics (FIGO) published a classification system based on the location of the myoma, which is used worldwide to describe different types of myomas [1].

• Types 0, 1 and 2: Submucosal fibroids are located just below the endometrium and protrude into the uterine cavity. Sometimes, they can be pedunculated in the cavity.
• Types 3 and 4: Intramural fibroids are located in the uterine wall. If they are of a certain size, they can contact the endometrium.
• Types 5, 6 and 7: Subserosal fibroids protrude on the serosal surface. Sometimes, they can be pedunculated in the pelvis.
• Type 8: Other locations, for example, cervical fibroids.
• Hybrid type: A fibroid which impacts both the endometrium and serosa.

Myomas can cause abnormal uterine bleeding, pelvic pain and reproductive dysfunction, although 50%–80% of affected women are asymptomatic [4–6].

The decision to start treatment for uterine myomas should be discussed with every patient and depends on the size and location of the myomas, the age of the patient, the symptoms of the patient and the desire to preserve fertility [7–9].

In asymptomatic patients, expectant management through watchful waiting can be discussed and considered [7].

Medical options include tranexamic acid and nonsteroidal anti‐inflammatory drugs (NSAID). Hormonal treatment options include the levonorgestrel‐releasing intrauterine device, oral contraceptives and gonadotropin‐releasing hormone (GnRH) agonists and antagonists. GnRH agonist and antagonist are primarily used preoperatively. They can reduce myoma size and bleeding but carry hypo‐oestrogenic side effects [7–9].

Surgical options encompass myomectomy, hysterectomy and uterine artery embolization. Myomectomy is the preferred surgical treatment option for patients wishing to preserve their fertility. Hysteroscopic myomectomy is an endoscopic procedure to remove submucosal (FIGO types 0, 1 and some type 2) myomas. The laparoscopic/robotic or laparotomic approach is indicated for the removal of intramural or subserosal myomas [7–9].

Pregnancy after myomectomy is generally safe, but the most feared complication is a uterine rupture, a rare but life‐threatening event for both mother and foetus. Earlier studies reported an incidence of uterine rupture after myomectomy during pregnancy of 0.60% [10]–0.75% [11]. In 2014, a systematic review and meta‐analysis of Claeys et al. found a risk of uterine rupture during pregnancy and labour of 0.75%. There was no significant difference between the risk of uterine rupture in laparoscopic (1.2%) and laparotomic (0.4%) myomectomy [11]. The risk during trial of labour after myomectomy (TOLAM) remains unclear. Potential risk factors for uterine rupture, such as surgical approach, haemostasis, suture technique, opening of the uterine cavity, myoma location and myoma type, have been discussed in the literature, but good evidence for a correlation is often lacking due to limited and heterogeneous data [10].

Consequently, there is no consensus regarding optimal management and mode of delivery in these patients. Most clinicians support TOLAM: 71%–79% after laparoscopic myomectomy and 75%–76% after laparotomic myomectomy [12–14]. This percentage is lower if the cavity is entered (14% and 27%, respectively) [12]. Other frequently mentioned factors that influence the decision to allow vaginal delivery are the number and location of the uterine incisions [12, 14]. As a result, there is a lack of national and international guidelines about whether TOLAM is safe, or in which situations it can be safe [15, 16].

We thus aimed to conduct an updated review of all available current literature on TOLAM outcomes in order to evaluate the current evidence and inform clinical decision‐making on this topic. The primary outcome of this systematic review is the risk of uterine rupture during TOLAM. The secondary outcomes were (1) the overall risk of uterine rupture during pregnancy and labour after myomectomy and (2) potential risk factors for uterine rupture after myomectomy irrespective of delivery mode. The present review includes only studies published after the review by Claeys et al., in order to provide an updated overview of the most recent evidence.

## 2. Materials and Methods

### 2.1. Research Question

We formulated a research question using the PICO framework (Population–Intervention–Comparison–Outcome): ‘What is the risk of uterine rupture during TOLAM?’

### 2.2. Search Strategy

A comprehensive search was conducted in MEDLINE (PubMed), Embase, Web of Science and Cochrane Library.

The search terms were as follows:

- Population: ‘myoma’, ‘myomatosis’, ‘myomatous’, ‘myomatectomy’, ‘myomectomy’, ‘myomotomy’, ‘fibroleiomyoma’, ‘fibroma’, ‘leiomyofibroma’, ‘fibroid’, ‘fibromyoma’, ‘leiomyoma’, ‘leiomyomata’, ‘leiomyomatous’
- Intervention: ‘delivery’, ‘birth’, ‘caesarean’, ‘fetectomy’, ‘ceasarotomy’, ‘section’, ‘sectio’, ‘labour’, ‘parturition’, ‘parturitional’
- Outcomes: ‘complication’, ‘birth’, ‘labour’, ‘peripartum’, ‘hysterorrhexis’, ‘metrorrhexis’, ‘uterine rupture’

For all these terms, we also used plurals and other spelling. A combination of the abovementioned terms was entered in the databases. The more detailed search strategy can be found in Supporting file 1.

We used restrictions on language and publication date. Articles published in English, French or Dutch and published between 1 April 2013 and 31 October 2023 were included.

By this systematic search, we found 1988 articles, which were imported in EndNote. Duplicates (541) were removed. The remaining 1447 articles were imported in Rayyan for further screening.

A second search for duplicates was done in Rayyan, by which another 26 articles were removed. The remaining articles were screened by two reviewers (CG and LP), independently of each other, on title and abstract. Restrictions were made on the type of article. Systematic reviews, meta‐analyses, literature reviews, surveys, case reports, case series and letters to editor were excluded. Articles not relevant to this systematic review were excluded (no myomectomy in medical history and/or no information about possible ruptures).

After removal of the excluded articles, 115 articles were sought for retrieval. For 71 articles, the full text could not be found. A total of 44 articles were screened in full text by 2 reviewers (CG and LP), independently of each other. Some articles were excluded after reading the full text because of publication type (reviews) and subject not relevant to this systematic review (no myomectomy in medical history and/or no information about possible ruptures).

Disagreements were resolved by discussion and if not possible by arbitration of a third reviewer (GV).

One additional article, that met the inclusion criteria, was included by the snowball method.

A total of 31 articles were included in this systematic review. Figure 1 presents a Preferred Reporting Items for Systematic Reviews and Meta‐Analyses (PRISMA) flow diagram of the abovementioned search strategy.

**FIGURE 1:**
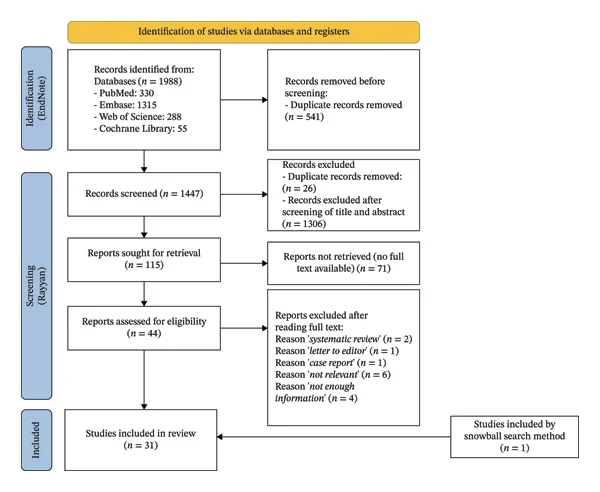
PRISMA flow diagram [17].

### 2.3. Data Extraction

Data from the included articles were summarised in an Excel spreadsheet. The following information was extracted: study design, type of myomectomy, number and size of myomas removed, location of the myomas removed, if the myomectomy involved entry into the uterine cavity, method of suturing of the myomectomy, time to pregnancy after myomectomy, weeks of pregnancy at time of rupture, location of rupture, mode of delivery, use of oxytocin and/or prostaglandins and maternal and foetal outcomes.

Miscarriages, ectopic pregnancies, termination of pregnancies (TOP), ongoing pregnancies and pregnancies with an unknown outcome were excluded.

When clarification was required, study authors were contacted for additional information.

### 2.4. Definitions

Because the classification of caesarean sections varies across studies, definitions were standardised for this review.

Primary caesarean section (PCS) was defined as an elective caesarean section performed on the scheduled date, before the onset of labour and with intact membranes.

Secondary caesarean section (SCS) was defined as any other caesarean section, including those performed after the onset of labour or rupture of membranes, or when a planned caesarean was advanced due to the start of labour.

### 2.5. Statistical Analysis

Categorical variables were analysed using Fisher’s exact test to compare patients with and without uterine rupture. Fisher’s exact test was chosen over the chi‐squared test because of the low number of events (uterine rupture), which resulted in expected cell counts below five in several contingency tables.

Continuous variables were assessed for normality using the Shapiro–Wilk test. As the data were not normally distributed, the Mann–Whitney *U* test was used for between‐group comparisons.

The overall incidence of uterine rupture was calculated as the number of ruptures divided by the total number of pregnancies after myomectomy, with an exact (Clopper–Pearson) 95% confidence interval.

Statistical analyses were performed using IBM SPSS Statistics Version 29 (IBM Corp., Armonk, NY, USA). A two‐sided *p* value < 0.05 was considered statistically significant.

## 3. Results

### 3.1. Results From Literature Search and Data Extraction

Table 1 lists the 31 articles that were included in this systematic review. A list is made of all pregnancies after previous myomectomies (13,381) and the number of ruptures (40) reported in these articles. The table shows a brief overview of the most relevant and available information extracted from the included articles.

**TABLE 1:** Overview of the included articles.

Publication year	Author	# pregnancies (*n*) (^∗^)	# ruptures		Twin		Type of myomectomy		Opening cavity		Methods of suturing		Induction of labour/use oxytocin/use prostaglandins		Modus partus	
			NR	R	NR	R	NR	R	NR	R	NR	R	NR	R	NR	R
2013	Fukuda et al. [18]	105	105	0	0		48 LSC, 57 LT		n.m.		1–4 layers		n.m.		33 VBAM, 58 PCS, 14 SCS	
2013	Kim et al. [19]	53	53	0	n.m.		44 LSC, 9 LT		n.m.		1‐2 layers		n.m.		3 VBAM, 47 PCS, 3 SCS	
2014	Fagherazzi et al. [20]	111	111	0	4		LSC		n.m.		1‐2‐3 layers		n.m.		42 VBAM, 69 CNS	
2014	Bernardi et al. [21]	39	35	4	n.m.	1	LSC	LSC	n.m.	2 Y, 2 N	1‐2 layers	1‐2 layers	n.m.	n.m.	13 VBAM, 16 PCS, 6 SCS	SCS
2015	Tian et al. [22]	106	106	0	n.m.		61 LSC, 45 LT		n.m.		1‐2 layers		n.m.		62 VBAM, 44 CNS	
2015	Pundir et al. [23]	18	18	0	n.m.		LT		n.m.		n.m.		n.m.		4 VBAM, 14 CNS	
2015	Floss et al. [24]	67	67	0	n.m.		LT		n.m.		2‐3 layers		n.m.		19 VBAM, 40 PCS, 8 SCS	
2015	Villot et al. [25]	18	18	0	0		17 LSC, 1 LT		5 Y, 13 N		1‐2 layers		1 induction, 2 use oxytocin		6 VBAM, 2 IVD, 8 PCS, 2 SCS	
2016	Chan et al. [26]	26	25	1	0	0	LSC	LSC	n.m.	n.m.	2 layers	2 layers	n.m.	n.m.	CNS	n.m.
2018	Gambacorti‐Passerini et al. [27]	110	110	0	0		70 LSC, 40 LT		9 Y, 88 N, 13 n.m.		n.m.		20 inductions		66 VBAM, 24 PCS, 9 SCS, 11 CNS	
2018	Kundu et al. [28]	114	114	0	0		17 LSC, 97 LT		n.m.		n.m.		n.m.		32 VBAM, 10 IVD 46 PCS, 26 SCS	
2019	Huberlant et al. [29]	24	24	0	0		robotic		n.m.		2‐3 layers		n.m.		7 VBAM, 17 CNS	
2020	Wu et al. [30]	173	173	0	0		LSC		n.m.		n.m.		n.m.		63 VBAM, 15 IVD, 95 CNS	
2020	Prapas et al. [31]	137	137	0	1		LSC		n.m.		1 layer		n.m.		10 VBAM, 127 CNS	
2020	La Verde et al. [32]	80	80	0	0		55 LSC, 25 LT		2 Y, 78 N		n.m.		26 inductions		50 VBAM, 4 IVD, 26 SCS	
2020	Kasuga et al. [33]	24	24	0	0		HSC		n.m.		n.m.		n.m.		11 VBAM, 3 IVD, 10 CNS	
2020	Pepin et al. [34]	60	60	0	n.m.		LSC		n.m.		n.m.		n.m.		6 VBAM, 54 CNS	
2020	Kim et al. [35]	89	88	1	88	1	n.m.	LSC	n.m.	n.m.	n.m.	n.m.	n.m.	n.m.	n.m.	SCS
2020	Lee et al. [36]	9890	9868	22	n.m.	n.m.	n.m.	n.m.	n.m.	n.m.	n.m.	n.m.	n.m.	n.m.	n.m.	n.m.
2020	Arena et al. [37]	70	70	0	n.m.		LSC		n.m.		1‐2 layers		n.m.		13 VBAM, 57 CNS	
2021	Hong et al. [38]	39	39	0	2		LSC		n.m.		n.m.		n.m.		3 VBAM, 36 CNS	
2021	Jiang et al. [39]	101	101	0	2		LSC		n.m.		n.m.		n.m.		18 VBAM, 83 CNS	
2021	Aksin et al. [40]	24	24	0	1		LSC		n.m.		1 layer		n.m.		PCS	
2022	Wada et al. [41]	180	178	2	n.m.	n.m.	LSC	LSC	25 Y, 153 N	N	n.m.	n.m.	n.m.	n.m.	CNS	CNS
2022	Don et al. [42]	115	114	1	n.m.	0	n.m.	n.m.	n.m.	Y	n.m.	n.m.	n.m.	n.m.	42 VBAM, 3 IVD, 49 PCS, 20 SCS	SCS
2022	Lu et al. [43]	105	105	0	n.m.		LSC		n.m.		n.m.		n.m.		n.m.	
2022	Ordás et al. [44]	52	52	0	0		31 LSC, 21 LT		n.m.		1‐2 layers		n.m.		20 VBAM, 23 PCS, 9 SCS	
2022	Kim et al. [45]	248	247	1	18	0	n.m.	n.m.	n.m.	n.m.	n.m.	n.m.	n.m.	n.m.	15 VBAM, 232 CNS	CNS
2023	Babunashvili et al. [46]	24	24	0	0		LT		n.m.		2 layers		n.m.		13 VBAM, 11 CNS	
2023	Won et al. [47]	182	181	1	n.m.	n.m.	127 LSC, 54 robotic	LSC	n.m.	n.m.	n.m.	n.m.	n.m.	n.m.	CNS	CNS
2023	Komatsu et al. [48]	997	990	7	0	0	590 LSC, 400 LT	6 LSC, 1 LT	n.m.	n.m.	n.m.	n.m.	n.m.	n.m.	71 VBAM, 91 IVD, 828 CNS	CNS
Total		13,381	13,341	40												

*Note:* R, group ‘ruptures’; NR, group ‘no ruptures’; Y, yes; N, no; LSC, laparoscopic; LT, laparotomic; HSC, hysteroscopic; CNS, caesarean section not specified.

Abbreviations: IVD, instrumental vaginal delivery; n.m., not mentioned; PCS, primary caesarean section; SCS, secondary caesarean section; VBAM, vaginal birth after myomectomy.

^∗^Number of pregnancies in patients with a myomectomy in the medical history (exclusion of miscarriage, ectopic pregnancy, TOP, unknown outcome of pregnancy and ongoing pregnancies).

Table 2 lists the 40 ruptures found in the included articles. All available information about the ruptures is shown in the presented table.

**TABLE 2:** Overview of all uterine ruptures after myomectomy.

Author	Twin	Type of myomectomy	Number of myomas removed	Size of myoma (cm)	Type of myoma	Cavity entry	Methods of suturing	Time to pregnancy (months)	Modus partus	Reason for CS	Gestational age at time of rupture (weeks)	Rupture without labour	Location rupture	Neonatal outcome
Bernardi et al. [21]	N	LSC	n.m.	≥ 4	Intramural or transmural	In 2/4 ruptures, unclear which one	1 or 2 layers	n.m.	SCS	n.m.	37	n.m.	n.m.	n.m.
Bernardi et al. [21]	N	LSC	n.m.	≥ 4	Intramural or transmural	In 2/4 ruptures, unclear which one	1 or 2 layers	n.m.	SCS	n.m.	40	n.m.	n.m.	n.m.
Bernardi et al. [21]	N	LSC	n.m.	≥ 4	Intramural or transmural	In 2/4 ruptures, unclear which one	1 or 2 layers	n.m.	SCS	Nonreassuring cardiotocographic monitoring	30	n.m.	n.m.	n.m.
Bernardi et al. [21]	Y	LSC	1	4	Intramural or transmural	In 2/4 ruptures, unclear which one	1 or 2 layers	n.m.	SCS	Nonreassuring cardiotocographic monitoring	24	Preterm labour after car accident	Posterior wall, location of the myomectomy	Perinatal death
Chan et al. [26]	N	LSC	n.m.	n.m.	n.m.	n.m.	2 layers	n.m.	n.m.	n.m.	24	n.m.	n.m.	Perinatal death
Kim et al. [35]	Y	LSC	2	3,5 and 2,8	2 intramural	n.m.	n.m.	7	SCS	Nonreassuring cardiotocographic monitoring	31	Hospitalized with preterm labour	Posterior wall, location of the myomectomy	n.m.
Lee et al. [36]	n.m.	n.m.	n.m.	n.m.	n.m.	n.m.	n.m.	n.m.	n.m.	n.m.	n.m.	n.m.	n.m.	n.m.
Lee et al. [36]	n.m.	n.m.	n.m.	n.m.	n.m.	n.m.	n.m.	n.m.	n.m.	n.m.	n.m.	n.m.	n.m.	n.m.
Lee et al. [36]	n.m.	n.m.	n.m.	n.m.	n.m.	n.m.	n.m.	n.m.	n.m.	n.m.	n.m.	n.m.	n.m.	n.m.
Lee et al. [36]	n.m.	n.m.	n.m.	n.m.	n.m.	n.m.	n.m.	n.m.	n.m.	n.m.	n.m.	n.m.	n.m.	n.m.
Lee et al. [36]	n.m.	n.m.	n.m.	n.m.	n.m.	n.m.	n.m.	n.m.	n.m.	n.m.	n.m.	n.m.	n.m.	n.m.
Lee et al. [36]	n.m.	n.m.	n.m.	n.m.	n.m.	n.m.	n.m.	n.m.	n.m.	n.m.	n.m.	n.m.	n.m.	n.m.
Lee et al. [36]	n.m.	n.m.	n.m.	n.m.	n.m.	n.m.	n.m.	n.m.	n.m.	n.m.	n.m.	n.m.	n.m.	n.m.
Lee et al. [36]	n.m.	n.m.	n.m.	n.m.	n.m.	n.m.	n.m.	n.m.	n.m.	n.m.	n.m.	n.m.	n.m.	n.m.
Lee et al. [36]	n.m.	n.m.	n.m.	n.m.	n.m.	n.m.	n.m.	n.m.	n.m.	n.m.	n.m.	n.m.	n.m.	n.m.
Lee et al. [36]	n.m.	n.m.	n.m.	n.m.	n.m.	n.m.	n.m.	n.m.	n.m.	n.m.	n.m.	n.m.	n.m.	n.m.
Lee et al. [36]	n.m.	n.m.	n.m.	n.m.	n.m.	n.m.	n.m.	n.m.	n.m.	n.m.	n.m.	n.m.	n.m.	n.m.
Lee et al. [36]	n.m.	n.m.	n.m.	n.m.	n.m.	n.m.	n.m.	n.m.	n.m.	n.m.	n.m.	n.m.	n.m.	n.m.
Lee et al. [36]	n.m.	n.m.	n.m.	n.m.	n.m.	n.m.	n.m.	n.m.	n.m.	n.m.	n.m.	n.m.	n.m.	n.m.
Lee et al. [36]	n.m.	n.m.	n.m.	n.m.	n.m.	n.m.	n.m.	n.m.	n.m.	n.m.	n.m.	n.m.	n.m.	n.m.
Lee et al. [36]	n.m.	n.m.	n.m.	n.m.	n.m.	n.m.	n.m.	n.m.	n.m.	n.m.	n.m.	n.m.	n.m.	n.m.
Lee et al. [36]	n.m.	n.m.	n.m.	n.m.	n.m.	n.m.	n.m.	n.m.	n.m.	n.m.	n.m.	n.m.	n.m.	n.m.
Lee et al. [36]	n.m.	n.m.	n.m.	n.m.	n.m.	n.m.	n.m.	n.m.	n.m.	n.m.	n.m.	n.m.	n.m.	n.m.
Lee et al. [36]	n.m.	n.m.	n.m.	n.m.	n.m.	n.m.	n.m.	n.m.	n.m.	n.m.	n.m.	n.m.	n.m.	n.m.
Lee et al. [36]	n.m.	n.m.	n.m.	n.m.	n.m.	n.m.	n.m.	n.m.	n.m.	n.m.	n.m.	n.m.	n.m.	n.m.
Lee et al. [36]	n.m.	n.m.	n.m.	n.m.	n.m.	n.m.	n.m.	n.m.	n.m.	n.m.	n.m.	n.m.	n.m.	n.m.
Lee et al. [36]	n.m.	n.m.	n.m.	n.m.	n.m.	n.m.	n.m.	n.m.	n.m.	n.m.	n.m.	n.m.	n.m.	n.m.
Lee et al. [36]	n.m.	n.m.	n.m.	n.m.	n.m.	n.m.	n.m.	n.m.	n.m.	n.m.	n.m.	n.m.	n.m.	n.m.
Wada et al. [41]	n.m.	LSC	n.m.	n.m.	n.m.	N	n.m.	n.m.	CNS	n.m.	n.m.	n.m.	n.m.	n.m.
Wada et al. [41]	n.m.	LSC	n.m.	n.m.	n.m.	N	n.m.	n.m.	CNS	n.m.	n.m.	n.m.	n.m.	n.m.
Don et al. [42]	N	LSC and HSC	4	The largest was 5	2 intramural and 2 subserosal	Y	n.m.	15	SCS	Advise for CS after myomectomy	35	Preterm labour	n.m.	Healthy child
Kim et al. [45]	N	n.m.	n.m.	n.m.	n.m.	n.m.	n.m.	n.m.	CNS	n.m.	n.m.	n.m.	n.m.	n.m.
Won et al. [47]	n.m.	LSC	n.m.	n.m.	n.m.	n.m.	n.m.	n.m.	CNS	n.m.	n.m.	n.m.	n.m.	n.m.
Komatsu et al. [48]	N	LSC	n.m.	n.m.	n.m.	n.m.	n.m.	n.m.	CNS	n.m.	n.m.	n.m.	n.m.	n.m.
Komatsu et al. [48]	N	LSC	n.m.	n.m.	n.m.	n.m.	n.m.	n.m.	CNS	n.m.	n.m.	n.m.	n.m.	n.m.
Komatsu et al. [48]	N	LSC	n.m.	n.m.	n.m.	n.m.	n.m.	n.m.	CNS	n.m.	n.m.	n.m.	n.m.	n.m.
Komatsu et al. [48]	N	LSC	n.m.	n.m.	n.m.	n.m.	n.m.	n.m.	CNS	n.m.	n.m.	n.m.	n.m.	n.m.
Komatsu et al. [48]	N	LSC	n.m.	n.m.	n.m.	n.m.	n.m.	n.m.	CNS	n.m.	n.m.	n.m.	n.m.	n.m.
Komatsu et al. [48]	N	LSC	n.m.	n.m.	n.m.	n.m.	n.m.	n.m.	CNS	n.m.	n.m.	n.m.	n.m.	n.m.
Komatsu et al. [48]	N	LT	n.m.	n.m.	n.m.	n.m.	n.m.	n.m.	CNS	n.m.	n.m.	n.m.	n.m.	n.m.

*Note:* Y, yes; N, no; LSC, laparoscopic; LT, laparotomic; HSC, hysteroscopic; CNS, caesarean section not specified.

Abbreviations: CS, caesarean section; n.m., not mentioned; PCS, primary caesarean section; SCS, secondary caesarean section.

### 3.2. Primary Outcome: The Risk of Uterine Rupture During TOLAM

None of the included studies provided sufficient information on the number of women who underwent a TOLAM. Several studies reported cases of successful vaginal delivery, but for most women who delivered by caesarean section, it was not possible to determine whether a trial of labour had been attempted. Among the 40 reported cases of uterine rupture, three occurred during labour. For the remaining cases, the available data were insufficient to establish whether the rupture took place during labour or before its onset.

Table 3 describes the number of patients with or without a uterine rupture and specifies the type of delivery. Of the 750 women who gave birth vaginally, none had a uterine rupture. Of the 2547 women who delivered through caesarean section, 17 women had a uterine rupture.

**TABLE 3:** The number of patients with or without a uterine rupture and the type of delivery.

Type of delivery	Rupture	No rupture	Total
VBAM	0	622	622
IVD	0	128	128
PCS	0	335	335
SCS	6	123	129
CNS	11	2072	2083
Not specified	23	10,061	10,084
Total	40	13,341	13,381

*Note:* CNS, caesarean section not specified.

Abbreviations: IVD, instrumental vaginal delivery; PCS, primary caesarean section; SCS, secondary caesarean section; VBAM, vaginal birth after myomectomy.

### 3.3. Secondary Outcomes

#### 3.3.1. Overall Risk of Uterine Rupture

A total of 13,381 patients who fulfilled a pregnancy after myomectomy were included. Forty of them had a uterine rupture during the pregnancy or during labour. Based on this literature review, the estimated overall risk of uterine rupture in pregnant patients with a history of a myomectomy is 0.30% (40/13,381; 95% CI: 0.21–0.41).

The gestational age at time of uterine rupture is known for 7 of the 40 ruptures. Five of the 7 uterine ruptures were preterm (gestational age of 24, 24, 30, 31 and 35 weeks), one rupture was at 37 weeks and one at 40 weeks of pregnancy. We conclude that at least 5 of the 7 ruptures would not have been prevented with a PCS given their preterm gestational age.

#### 3.3.2. Uterine Rupture and Type of Myomectomy

Table 4 describes the number of patients with or without a uterine rupture and type of myomectomy.

**TABLE 4:** The number of patients with or without a uterine rupture and the type of myomectomy.

Type of myomectomy	Rupture	No rupture	Total
LT	1	804	805
LSC	15	2118	2133
HSC	0	24	24
Robotic	0	78	78
Not specified	24	10,317	10,341
Total	40	13,341	13,381

*Note:* LT, laparotomic; LSC, laparoscopic; HSC, hysteroscopic.

The risk of uterine rupture appeared to be higher after laparoscopic myomectomy (15/2133 or 0.70%) compared with laparotomic myomectomy (1/805 or 0.12%). However, this difference was not statistically significant (*p* = 0.09) when using Fisher’s exact test.

No significant differences were found between the other types of myomectomies, as these groups were small and no events occurred.

#### 3.3.3. Uterine Rupture and Opening of the Cavity

Table 5 describes the number of patients with or without a uterine rupture and whether the cavity was opened during the myomectomy.

**TABLE 5:** The number of patients with or without a uterine rupture and whether the cavity was opened during the myomectomy.

Opening of the cavity	Rupture	No rupture	Total
Yes	3	41	44
No	4	332	336
Not specified	33	12,968	13,001
Total	40	13,341	13,381

In the subset of women for whom surgical details were reported (*n* = 380), uterine rupture was more frequently observed when the uterine cavity had been entered during myomectomy (3/44; 6.82%) than when it had not (4/336; 1.19%) (Fisher’s exact test OR: 6.07; 95% CI: 1.31–28.10; *p* = 0.04).

#### 3.3.4. Other Risk Factors

The included articles did not provide enough detailed information about other potential risk factors, such as the size, location and number of myomas removed, the suturing method and the time between myomectomy and pregnancy, preventing reliable assessment of their association with uterine rupture.

## 4. Discussion

### 4.1. Main Findings

This systematic review aimed to report the incidence of uterine rupture during TOLAM. We were unable to measure this primary outcome as the included studies did not explicitly distinguish between uterine rupture before or during labour.

### 4.2. Comparison With Literature

The overall incidence of uterine rupture in pregnancies following a prior myomectomy identified in this systematic review (0.30%) is slightly lower than the incidence reported in previous literature, which ranged from 0.60% [10] to 0.75% [11]. This study is an update of the past 10 years of research, during which advances in surgical techniques, including the increased use of laparoscopic approaches, improved haemostatic control and refined suturing methods, may have contributed to a reduced risk of uterine rupture [49, 50].

### 4.3. Cavity Entry as Risk Factor

This analysis suggests a higher incidence of uterine rupture after myomectomy when the uterine cavity was entered. This finding is based on a small number of women and events (7 ruptures among 380 patients) and should be interpreted with caution. The available data are limited by potential selection and reporting biases, lack of adjustment for confounders (for example, myoma size, location and suture technique) and substantial missing information for the majority of the cohort. Therefore, while cavity entry may be an important risk marker and warrants further study, these results are insufficient to establish causality or to support changes in clinical practice. This risk factor was not assessed in the reviews of Claeys et al. and Gambacorti‐Passerini et al. [10, 11].

### 4.4. Other Risk Factors

Reviewing the current literature, we noticed that studies are inconclusive regarding risk factors for uterine rupture after myomectomy. In our systematic review, laparoscopic myomectomy appeared to carry a higher risk of uterine rupture compared with other types of myomectomy, but this was not statistically significant (*p* = 0.09). This is similar to the reviews of Claeys et al. and Gambacorti‐Passerini et al., where it was also not possible to determine whether myomectomy surgical approach is a significant risk factor. In the study by Ginod et al., a higher risk of uterine rupture was observed in the laparoscopic myomectomy group compared to the laparotomic myomectomy group [51].

The location and size(s) of the myomas and suturing technique used during the myomectomy have also been suggested as possible risk factors, but neither our review nor those of Claeys et al. and Gambacorti‐Passerini et al. were able to confirm this [10, 11].

### 4.5. Comparison With Trial of Labour After Caesarean Section (TOLAC)

Counselling of women with a uterine scar is often informed by the extensive literature on TOLAC, where the risk of uterine rupture is generally reported around 0.2%–0.69% [47, 48]. In contrast, data specific to TOLAM remain limited and heterogeneous, with inconsistent reporting of whether labour was attempted. Nevertheless, the overall rupture risk after myomectomy in our review appears to be of the same order of magnitude as that observed during TOLAC, suggesting that pregnancy after myomectomy does not confer an obviously higher risk of rupture than pregnancy with a prior caesarean scar. This comparison should be interpreted with caution, however, as the pathophysiology of the uterine scar differs between caesarean section and myomectomy, and direct extrapolation of TOLAC data to the post‐myomectomy setting may not be appropriate.

### 4.6. Strengths and Limitations

The main strength of this systematic review is the large study population that is included in our study. This provides a realistic incidence of uterine rupture after a myomectomy. Second, by restricting inclusion to the period 2013–2023, this review specifically captures the most recent decade of surgical practice, making it a true update of the earlier systematic reviews by Claeys et al. [11] and Gambacorti‐Passerini et al. [10]. Third, article screening was performed independently by two reviewers, reducing the risk of selection bias. Finally, standardised definitions for PCS and SCS were applied across all included studies, which improves consistency in the classification of delivery mode.

Our review has several limitations. First, there is a high risk of publication bias. We included some large studies (more than half of the uterine ruptures found in our literature review originate from the study of Lee et al. [36]) with a good study design, but they did not mention the characteristics of the myomectomy, the delivery and the rupture itself. We contacted the authors of the included articles in this systematic review by mail to obtain individual patient data, but unfortunately without success.

Many healthcare professionals prefer to perform an elective caesarean section in patients with a history of a myomectomy. This inevitably causes a risk of performance bias, as these patients did not go through labour and therefore may have a lower chance of uterine rupture in comparison to patients with TOLAM.

We intended to separately assess possible high‐risk groups, e.g., multiple pregnancies and patients with a history of uterine surgery (other than the myomectomy) from the general population in our calculations. However, we did not have enough information to carry out this subanalysis.

We chose to exclude case reports and case series to obtain the most realistic incidence possible. As a result, however, we might have missed important information about characteristics and possible risk factors of uterine rupture after a previous myomectomy.

### 4.7. Future Research

As mentioned above, many included studies lacked information on the characteristics of the myomectomy, the delivery and the rupture itself. This makes it difficult to provide definitive and comprehensive clinical guidance based on the available literature for this patient group. There is thus a need for good randomised controlled trials (RCTs). The rare occurrence of uterine rupture implies that there is a need for a large multicentre study which compares TOLAM versus a PCS. All characteristics of the myomectomy, the delivery and the rupture itself should be recorded. Only then, we will be able to make inferences about risk factors for uterine rupture after a previous myomectomy. An RCT would arguably not be ethically feasible due to unclear risks of TOLAM, and the low absolute incidence of uterine rupture would require a restrictively large sample size.

## 5. Conclusion

In conclusion, based on this literature review, the estimated overall incidence of uterine rupture in pregnant patients after a previous myomectomy is 0.30%. We cannot report an estimated incidence of uterine rupture during TOLAM, due to a lack of specific data.

Entry into the uterine cavity during myomectomy was associated with a significantly higher risk of uterine rupture (OR: 6.07; *p* = 0.04), although this finding is based on a small subset and should be confirmed in larger studies. No other risk factors, including surgical approach, could be reliably identified.

The overall rupture risk after myomectomy appears to be of the same order of magnitude as that reported during TOLAC (0.2%–0.69%), suggesting that pregnancy after myomectomy does not confer an obviously higher risk than pregnancy with a prior caesarean scar. Because the majority of ruptures occurred before the onset of labour, routine elective prelabour caesarean delivery is unlikely to prevent all events and should not be regarded as a universally safer strategy. For women without cavity entry and without additional major risk factors, offering TOLAM may be reasonable, as the absolute risk of rupture seems low and broadly comparable to the risk accepted during TOLAC. Counselling should take place within a shared decision‐making framework that balances the small but serious risk of uterine rupture against the short‐ and long‐term consequences of repeat caesarean delivery, including implications for future pregnancies.

**Nomenclature**
CG: Camille Groffils
CI: Confidence interval
CNS: Caesarean section not specified
FIGO: International Federation of Gynaecology and Obstetrics
GnRH: Gonadotropin‐releasing hormone
GV: Griet Vandenberghe
HSC: Hysteroscopic
IVD: Instrumental vaginal delivery
LP: Lena Paelinck
LSC: Laparoscopic
LT: Laparotomic
n.m.: Not mentioned
NSAID: Nonsteroidal anti‐inflammatory drugs
PCS: Primary caesarean section
PICO: Population–Intervention–Comparison–Outcome
PRISMA: Preferred Reporting Items for Systematic Reviews and Meta‐Analyses
RCT: Randomised controlled trial
SCS: Secondary caesarean section
TOLAC: Trial of labour after caesarean section
TOLAM: Trial of labour after myomectomy
TOP: Termination of pregnancy
VBAM: Vaginal birth after myomectomy

## Funding

This research did not receive any specific grant from funding agencies in the public, commercial or not‐for‐profit sectors.

## Disclosure

This work was conducted while the authors were employed at Ghent University Hospital.

## Conflicts of Interest

The authors declare no conflicts of interest.

## Supporting Information

Additional supporting information can be found online in the Supporting Information section.

## Supporting information


**Supporting Information** Supporting file 1: Detailed search strategy.

## Data Availability

The data that support the findings of this study are available within the article and its supporting information. The dataset was generated by extracting information from previously published studies, which are cited in the reference list.
