# Supplementary material for: Systematic Review of Pregnancy After Myomectomy: The Risk of Uterine Rupture
Source: Obstet Gynecol Int. 2026 Oct 17;2026:6048559. doi: 10.1155/ogi/6048559 (PMC13676031; doi:10.1155/ogi/6048559)
Supplement: Supplementary file 1 [file OGI-2026-6048559-s001.docx]

# Supplementary materials

## Supplementary file 1: detailed search strategy

| **Research question (e.g. PICO format):**  P: pregnant patients with a myomectomy in their medical history  I: labour  C: -  O: risk of uterine rupture | | |
| --- | --- | --- |
| **Type of review (e.g. systematic review, scoping review, narrative review, etc.):** Systematic review | | |
| **NAME OF DATABASE (interface):** Pubmed | | |
| **Concept** | **Line number** | **Search strategy** |
| Concept 1: | 1 | **("myoma"[MeSH Terms] OR "myoma"[Title/Abstract] OR "myomas"[Title/Abstract] OR "myoma s"[Title/Abstract] OR ("leiomyoma"[MeSH Terms] OR "leiomyoma"[Title/Abstract] OR "leiomyomas"[Title/Abstract]) OR ("fibroid s"[Title/Abstract] OR "fibroid"[Title/Abstract] OR "fibroids"[Title/Abstract]) OR ("fibromyoma"[Title/Abstract] OR "fibromyomas"[Title/Abstract]) OR ("uterine myomectomy"[MeSH Terms] OR "myomectomies"[Title/Abstract] OR "myomectomy"[Title/Abstract] OR "myomectomys"[Title/Abstract]) OR "myomectomie"[Title/Abstract] OR "myomatosis"[Title/Abstract] OR "myomatous"[Title/Abstract] OR "myomatectomy"[Title/Abstract] OR "myomatectomie"[Title/Abstract] OR "myomatectomys"[Title/Abstract] OR "myomatectomies"[Title/Abstract] OR "myomotomy"[Title/Abstract] OR "myomotomie"[Title/Abstract] OR "myomotomys"[Title/Abstract] OR "myomotomies"[Title/Abstract] OR "fibroleiomyoma"[Title/Abstract] OR "fibroleiomyomas"[Title/Abstract] OR "fibroma"[Title/Abstract] OR "fibromas"[Title/Abstract] OR "leiomyofibroma"[Title/Abstract] OR "leiomyofibromas"[Title/Abstract] OR "leiomyomatous"[Title/Abstract] OR "leyomyoma"[Title/Abstract] OR "leyomyomas"[Title/Abstract] OR "fibro-myoma"[Title/Abstract] OR "fibro-myomas"[Title/Abstract] OR "leiomyomata"[Title/Abstract] OR "leiomyomatas"[Title/Abstract]) OR "leyomyofibroma"[Title/Abstract] OR "leyomyofibromas"[Title/Abstract] OR "leyomyomata"[Title/Abstract] OR "leyomyomatas"[Title/Abstract] OR "leyomyomatous"[Title/Abstract] OR "fibroleyomyoma"[Title/Abstract] OR "fibroleyomyomas"[Title/Abstract]** |
| Concept 2: | 2 | ("section"[Title/Abstract] OR "cesarean section"[MeSH Terms] OR "cesarean"[Title/Abstract] OR "cesareans"[Title/Abstract] OR "cesarian"[Title/Abstract] OR "cesarians"[Title/Abstract] OR "caesarean"[Title/Abstract] OR "caesareans"[Title/Abstract] OR "caesarian"[Title/Abstract] OR "caesarians"[Title/Abstract] OR "ceasarean"[Title/Abstract] OR "ceasareans"[Title/Abstract] OR "ceasarian"[Title/Abstract] OR "ceasarians"[Title/Abstract] OR ("sections"[Title/Abstract] OR “sectio"[Title/Abstract] OR “sectios"[Title/Abstract]) OR ("deliveries"[Title/Abstract] OR "delivery, obstetric"[MeSH Terms] OR "deliverie"[Title/Abstract] OR "deliverys"[Title/Abstract] OR "delivery"[Title/Abstract]) OR ("parturition"[MeSH Terms] OR "parturition"[Title/Abstract] OR "parturitions"[Title/Abstract] OR "parturitional"[Title/Abstract]) OR ("labour"[Title/Abstract] OR "trial of labor"[MeSH Terms] OR "labor"[Title/Abstract]) OR "labor, obstetric"[MeSH Terms]) OR "birth"[Title/Abstract] OR "fetectomy"[Title/Abstract] OR "fetectomie"[Title/Abstract] OR "fetectomys"[Title/Abstract] OR "fetectomies"[Title/Abstract] OR "cesarotomy"[Title/Abstract] OR "cesarotomie"[Title/Abstract] OR "cesarotomies"[Title/Abstract] OR "cesarotomys"[Title/Abstract] OR "caesarotomy"[Title/Abstract] OR "caesarotomie"[Title/Abstract] OR "caesarotomies"[Title/Abstract] OR "caesarotomys"[Title/Abstract] OR "ceasarotomy"[Title/Abstract] OR "ceasarotomie"[Title/Abstract] OR "ceasarotomies"[Title/Abstract] OR "ceasarotomys"[Title/Abstract] |
| Concept 3: | 3 | ("uterine rupture"[MeSH Terms] OR ("uterine"[Title/Abstract] AND "rupture"[Title/Abstract]) OR ("uterine"[Title/Abstract] AND "ruptures"[Title/Abstract]) OR ("uterus"[Title/Abstract] AND "rupture"[Title/Abstract]) OR ("uterus"[Title/Abstract] AND "ruptures"[Title/Abstract]) OR ("ruptured"[Title/Abstract] AND "uterus"[Title/Abstract]) OR "ruptura uteri"[Title/Abstract] OR "metrorrhexis"[Title/Abstract] OR "hysterorrhexis"[Title/Abstract] OR ("peri-partum"[Title/Abstract] AND "complication"[Title/Abstract]) OR ("peri-partum"[Title/Abstract] AND "complications"[Title/Abstract]) OR ("peripartum"[Title/Abstract] AND "complication"[Title/Abstract]) OR ("peripartum"[Title/Abstract] AND "complications"[Title/Abstract]) OR ("birth"[Title/Abstract] AND "complication"[Title/Abstract]) OR ("birth"[Title/Abstract] AND "complications"[Title/Abstract]) OR (("labour"[Title/Abstract] AND "complications"[Title/Abstract]) OR "obstetric labor complications"[MeSH Terms] OR ("labor"[Title/Abstract] AND "complication"[Title/Abstract])) OR (("labour"[Title/Abstract] AND "complication"[Title/Abstract]) OR ("labor"[Title/Abstract] AND "complications"[Title/Abstract]))) |
| Filter / search block: | 4 | / |
| Combination of concepts | 5 | 1 AND 2 AND 3  **(("myoma"[MeSH Terms] OR "myoma"[Title/Abstract] OR "myomas"[Title/Abstract] OR "myoma s"[Title/Abstract] OR ("leiomyoma"[MeSH Terms] OR "leiomyoma"[Title/Abstract] OR "leiomyomas"[Title/Abstract]) OR ("fibroid s"[Title/Abstract] OR "fibroid"[Title/Abstract] OR "fibroids"[Title/Abstract]) OR ("fibromyoma"[Title/Abstract] OR "fibromyomas"[Title/Abstract]) OR ("uterine myomectomy"[MeSH Terms] OR "myomectomies"[Title/Abstract] OR "myomectomy"[Title/Abstract] OR "myomectomys"[Title/Abstract]) OR "myomectomie"[Title/Abstract] OR "myomatosis"[Title/Abstract] OR "myomatous"[Title/Abstract] OR "myomatectomy"[Title/Abstract] OR "myomatectomie"[Title/Abstract] OR "myomatectomys"[Title/Abstract] OR "myomatectomies"[Title/Abstract] OR "myomotomy"[Title/Abstract] OR "myomotomie"[Title/Abstract] OR "myomotomys"[Title/Abstract] OR "myomotomies"[Title/Abstract] OR "fibroleiomyoma"[Title/Abstract] OR "fibroleiomyomas"[Title/Abstract] OR "fibroma"[Title/Abstract] OR "fibromas"[Title/Abstract] OR "leiomyofibroma"[Title/Abstract] OR "leiomyofibromas"[Title/Abstract] OR "leiomyomatous"[Title/Abstract] OR "leyomyoma"[Title/Abstract] OR "leyomyomas"[Title/Abstract] OR "fibro-myoma"[Title/Abstract] OR "fibro-myomas"[Title/Abstract] OR "leiomyomata"[Title/Abstract] OR "leiomyomatas"[Title/Abstract]) OR "leyomyofibroma"[Title/Abstract] OR "leyomyofibromas"[Title/Abstract] OR "leyomyomata"[Title/Abstract] OR "leyomyomatas"[Title/Abstract] OR "leyomyomatous"[Title/Abstract] OR "fibroleyomyoma"[Title/Abstract] OR "fibroleyomyomas"[Title/Abstract])** AND (("section"[Title/Abstract] OR "cesarean section"[MeSH Terms] OR "cesarean"[Title/Abstract] OR "cesareans"[Title/Abstract] OR "cesarian"[Title/Abstract] OR "cesarians"[Title/Abstract] OR "caesarean"[Title/Abstract] OR "caesareans"[Title/Abstract] OR "caesarian"[Title/Abstract] OR "caesarians"[Title/Abstract] OR "ceasarean"[Title/Abstract] OR "ceasareans"[Title/Abstract] OR "ceasarian"[Title/Abstract] OR "ceasarians"[Title/Abstract] OR ("sections"[Title/Abstract] OR “sectio"[Title/Abstract] OR “sectios"[Title/Abstract]) OR ("deliveries"[Title/Abstract] OR "delivery, obstetric"[MeSH Terms] OR "deliverie"[Title/Abstract] OR "deliverys"[Title/Abstract] OR "delivery"[Title/Abstract]) OR ("parturition"[MeSH Terms] OR "parturition"[Title/Abstract] OR "parturitions"[Title/Abstract] OR "parturitional"[Title/Abstract]) OR ("labour"[Title/Abstract] OR "trial of labor"[MeSH Terms] OR "labor"[Title/Abstract]) OR "labor, obstetric"[MeSH Terms]) OR "birth"[Title/Abstract] OR "fetectomy"[Title/Abstract] OR "fetectomie"[Title/Abstract] OR "fetectomys"[Title/Abstract] OR "fetectomies"[Title/Abstract] OR "cesarotomy"[Title/Abstract] OR "cesarotomie"[Title/Abstract] OR "cesarotomies"[Title/Abstract] OR "cesarotomys"[Title/Abstract] OR "caesarotomy"[Title/Abstract] OR "caesarotomie"[Title/Abstract] OR "caesarotomies"[Title/Abstract] OR "caesarotomys"[Title/Abstract] OR "ceasarotomy"[Title/Abstract] OR "ceasarotomie"[Title/Abstract] OR "ceasarotomies"[Title/Abstract] OR "ceasarotomys"[Title/Abstract]) AND (("uterine rupture"[MeSH Terms] OR ("uterine"[Title/Abstract] AND "rupture"[Title/Abstract]) OR ("uterine"[Title/Abstract] AND "ruptures"[Title/Abstract]) OR ("uterus"[Title/Abstract] AND "rupture"[Title/Abstract]) OR ("uterus"[Title/Abstract] AND "ruptures"[Title/Abstract]) OR ("ruptured"[Title/Abstract] AND "uterus"[Title/Abstract]) OR "ruptura uteri"[Title/Abstract] OR "metrorrhexis"[Title/Abstract] OR "hysterorrhexis"[Title/Abstract] OR ("peri-partum"[Title/Abstract] AND "complication"[Title/Abstract]) OR ("peri-partum"[Title/Abstract] AND "complications"[Title/Abstract]) OR ("peripartum"[Title/Abstract] AND "complication"[Title/Abstract]) OR ("peripartum"[Title/Abstract] AND "complications"[Title/Abstract]) OR ("birth"[Title/Abstract] AND "complication"[Title/Abstract]) OR ("birth"[Title/Abstract] AND "complications"[Title/Abstract]) OR (("labour"[Title/Abstract] AND "complications"[Title/Abstract]) OR "obstetric labor complications"[MeSH Terms] OR ("labor"[Title/Abstract] AND "complication"[Title/Abstract])) OR (("labour"[Title/Abstract] AND "complication"[Title/Abstract]) OR ("labor"[Title/Abstract] AND "complications"[Title/Abstract])))) |

| **Research question (e.g. PICO format):**  P: pregnant patients with a myomectomy in their medical history  I: labour  C: -  O: risk of uterine rupture | | |
| --- | --- | --- |
| **Type of review (e.g. systematic review, scoping review, narrative review, etc.):** Systematic review | | |
| **NAME OF DATABASE (interface):** Embase | | |
| **Concept** | **Line number** | **Search strategy** |
| Concept 1: | 1 | 'myoma'/exp OR 'myoma':ti,ab,kw OR 'myomatosis':ti,ab,kw OR 'myomatous':ti,ab,kw OR 'myomectomy'/exp OR 'myomectomie':ti,ab,kw OR 'myomectomies':ti,ab,kw OR 'myomatectomy':ti,ab,kw OR 'myomatectomie':ti,ab,kw OR 'myomatectomys':ti,ab,kw OR 'myomatectomies':ti,ab,kw OR 'myomectomy':ti,ab,kw OR 'myomectomys':ti,ab,kw OR 'myomotomy':ti,ab,kw OR 'myomotomie':ti,ab,kw OR 'myomotomys':ti,ab,kw OR 'myomotomies':ti,ab,kw OR 'fibroleiomyoma':ti,ab,kw OR 'fibroleiomyomas':ti,ab,kw OR 'fibroma':ti,ab,kw OR 'fibromas':ti,ab,kw OR 'fibromyomas':ti,ab,kw OR 'leiomyofibroma':ti,ab,kw OR 'leiomyofibromas':ti,ab,kw OR 'leiomyomas':ti,ab,kw OR 'myomas':ti,ab,kw OR 'fibro-myoma':ti,ab,kw OR 'fibro-myomas':ti,ab,kw OR 'fibroid':ti,ab,kw OR 'fibroids':ti,ab,kw OR 'fibromyoma':ti,ab,kw OR 'leiomyoma':ti,ab,kw OR 'leiomyomata':ti,ab,kw OR 'leiomyomatas':ti,ab,kw OR 'leiomyomatous':ti,ab,kw OR 'leyomyoma':ti,ab,kw OR 'leyomyomas':ti,ab,kw OR 'leyomyofibroma':ti,ab,kw OR 'leyomyofibromas':ti,ab,kw OR 'leyomyomata':ti,ab,kw OR 'leyomyomatas':ti,ab,kw OR 'leyomyomatous':ti,ab,kw OR 'fibroleyomyoma':ti,ab,kw OR 'fibroleyomyomas':ti,ab,kw |
| Concept 2: | 2 | 'obstetric delivery'/exp OR 'delivery':ti,ab,kw OR 'deliverys':ti,ab,kw OR 'deliveries':ti,ab,kw OR 'deliverie':ti,ab,kw OR 'cesarean section'/exp OR 'birth':ti,ab,kw OR 'caesarean':ti,ab,kw OR 'caesareans':ti,ab,kw OR 'cesarean':ti,ab,kw OR 'cesareans':ti,ab,kw OR 'ceasarean':ti,ab,kw OR 'ceasareans':ti,ab,kw OR 'caesarian':ti,ab,kw OR 'caesarians':ti,ab,kw OR 'cesarian':ti,ab,kw OR 'cesarians':ti,ab,kw OR 'ceasarian':ti,ab,kw OR 'ceasarians':ti,ab,kw OR 'fetectomy':ti,ab,kw OR 'fetectomie':ti,ab,kw OR 'fetectomys':ti,ab,kw OR 'fetectomies':ti,ab,kw OR 'cesarotomy':ti,ab,kw OR 'cesarotomie':ti,ab,kw OR 'cesarotomies':ti,ab,kw OR 'cesarotomys':ti,ab,kw OR 'caesarotomy':ti,ab,kw OR 'caesarotomie':ti,ab,kw OR 'caesarotomies':ti,ab,kw OR 'caesarotomys':ti,ab,kw OR 'ceasarotomy':ti,ab,kw OR 'ceasarotomie':ti,ab,kw OR 'ceasarotomies':ti,ab,kw OR 'ceasarotomys':ti,ab,kw OR 'section':ti,ab,kw OR 'sections':ti,ab,kw OR 'sectio':ti,ab,kw OR 'sectios':ti,ab,kw OR 'labor'/exp OR 'labor':ti,ab,kw OR 'labour':ti,ab,kw OR 'parturition':ti,ab,kw OR 'parturitions':ti,ab,kw OR 'parturitional':ti,ab,kw |
| Concept 3: | 3 | 'labor complication'/exp OR ('birth':ti,ab,kw AND 'complications':ti,ab,kw) OR ('birth':ti,ab,kw AND 'complication':ti,ab,kw) OR ('labor':ti,ab,kw AND 'complication':ti,ab,kw) OR ('labour':ti,ab,kw AND 'complication':ti,ab,kw) OR ('labor':ti,ab,kw AND 'complications':ti,ab,kw) OR ('labour':ti,ab,kw AND 'complications':ti,ab,kw) OR ('peri-partum':ti,ab,kw AND 'complication':ti,ab,kw) OR ('peri-partum':ti,ab,kw AND 'complications':ti,ab,kw) OR ('peripartum':ti,ab,kw AND 'complication':ti,ab,kw) OR ('peripartum':ti,ab,kw AND 'complications':ti,ab,kw) OR 'hysterorrhexis':ti,ab,kw OR 'metrorrhexis':ti,ab,kw OR 'ruptura uteri':ti,ab,kw OR ('rupture':ti,ab,kw AND 'uterus':ti,ab,kw) OR ('ruptured':ti,ab,kw AND 'uterus':ti,ab,kw) OR ('uterine':ti,ab,kw AND 'rupture':ti,ab,kw) OR ('ruptures':ti,ab,kw AND 'uterus':ti,ab,kw) OR ('uterine':ti,ab,kw AND 'ruptures':ti,ab,kw) |
| Filter / search block: | 4 | / |
| Combination of concepts | 5 | 1 AND 2 AND 3  ('myoma'/exp OR 'myoma':ti,ab,kw OR 'myomatosis':ti,ab,kw OR 'myomatous':ti,ab,kw OR 'myomectomy'/exp OR 'myomectomie':ti,ab,kw OR 'myomectomies':ti,ab,kw OR 'myomatectomy':ti,ab,kw OR 'myomatectomie':ti,ab,kw OR 'myomatectomys':ti,ab,kw OR 'myomatectomies':ti,ab,kw OR 'myomectomy':ti,ab,kw OR 'myomectomys':ti,ab,kw OR 'myomotomy':ti,ab,kw OR 'myomotomie':ti,ab,kw OR 'myomotomys':ti,ab,kw OR 'myomotomies':ti,ab,kw OR 'fibroleiomyoma':ti,ab,kw OR 'fibroleiomyomas':ti,ab,kw OR 'fibroma':ti,ab,kw OR 'fibromas':ti,ab,kw OR 'fibromyomas':ti,ab,kw OR 'leiomyofibroma':ti,ab,kw OR 'leiomyofibromas':ti,ab,kw OR 'leiomyomas':ti,ab,kw OR 'myomas':ti,ab,kw OR 'fibro-myoma':ti,ab,kw OR 'fibro-myomas':ti,ab,kw OR 'fibroid':ti,ab,kw OR 'fibroids':ti,ab,kw OR 'fibromyoma':ti,ab,kw OR 'leiomyoma':ti,ab,kw OR 'leiomyomata':ti,ab,kw OR 'leiomyomatas':ti,ab,kw OR 'leiomyomatous':ti,ab,kw OR 'leyomyoma':ti,ab,kw OR 'leyomyomas':ti,ab,kw OR 'leyomyofibroma':ti,ab,kw OR 'leyomyofibromas':ti,ab,kw OR 'leyomyomata':ti,ab,kw OR 'leyomyomatas':ti,ab,kw OR 'leyomyomatous':ti,ab,kw OR 'fibroleyomyoma':ti,ab,kw OR 'fibroleyomyomas':ti,ab,kw) AND ('obstetric delivery'/exp OR 'delivery':ti,ab,kw OR 'deliverys':ti,ab,kw OR 'deliveries':ti,ab,kw OR 'deliverie':ti,ab,kw OR 'cesarean section'/exp OR 'birth':ti,ab,kw OR 'caesarean':ti,ab,kw OR 'caesareans':ti,ab,kw OR 'cesarean':ti,ab,kw OR 'cesareans':ti,ab,kw OR 'ceasarean':ti,ab,kw OR 'ceasareans':ti,ab,kw OR 'caesarian':ti,ab,kw OR 'caesarians':ti,ab,kw OR 'cesarian':ti,ab,kw OR 'cesarians':ti,ab,kw OR 'ceasarian':ti,ab,kw OR 'ceasarians':ti,ab,kw OR 'fetectomy':ti,ab,kw OR 'fetectomie':ti,ab,kw OR 'fetectomys':ti,ab,kw OR 'fetectomies':ti,ab,kw OR 'cesarotomy':ti,ab,kw OR 'cesarotomie':ti,ab,kw OR 'cesarotomies':ti,ab,kw OR 'cesarotomys':ti,ab,kw OR 'caesarotomy':ti,ab,kw OR 'caesarotomie':ti,ab,kw OR 'caesarotomies':ti,ab,kw OR 'caesarotomys':ti,ab,kw OR 'ceasarotomy':ti,ab,kw OR 'ceasarotomie':ti,ab,kw OR 'ceasarotomies':ti,ab,kw OR 'ceasarotomys':ti,ab,kw OR 'section':ti,ab,kw OR 'sections':ti,ab,kw OR 'sectio':ti,ab,kw OR 'sectios':ti,ab,kw OR 'labor'/exp OR 'labor':ti,ab,kw OR 'labour':ti,ab,kw OR 'parturition':ti,ab,kw OR 'parturitions':ti,ab,kw OR 'parturitional':ti,ab,kw) AND ('labor complication'/exp OR ('birth':ti,ab,kw AND 'complications':ti,ab,kw) OR ('birth':ti,ab,kw AND 'complication':ti,ab,kw) OR ('labor':ti,ab,kw AND 'complication':ti,ab,kw) OR ('labour':ti,ab,kw AND 'complication':ti,ab,kw) OR ('labor':ti,ab,kw AND 'complications':ti,ab,kw) OR ('labour':ti,ab,kw AND 'complications':ti,ab,kw) OR ('peri-partum':ti,ab,kw AND 'complication':ti,ab,kw) OR ('peri-partum':ti,ab,kw AND 'complications':ti,ab,kw) OR ('peripartum':ti,ab,kw AND 'complication':ti,ab,kw) OR ('peripartum':ti,ab,kw AND 'complications':ti,ab,kw) OR 'hysterorrhexis':ti,ab,kw OR 'metrorrhexis':ti,ab,kw OR 'ruptura uteri':ti,ab,kw OR ('rupture':ti,ab,kw AND 'uterus':ti,ab,kw) OR ('ruptured':ti,ab,kw AND 'uterus':ti,ab,kw) OR ('uterine':ti,ab,kw AND 'rupture':ti,ab,kw) OR ('ruptures':ti,ab,kw AND 'uterus':ti,ab,kw) OR ('uterine':ti,ab,kw AND 'ruptures':ti,ab,kw)) |

| **Research question (e.g. PICO format):**  P: pregnant patients with a myomectomy in their medical history  I: labour  C: -  O: risk of uterine rupture | | |
| --- | --- | --- |
| **Type of review (e.g. systematic review, scoping review, narrative review, etc.):** Systematic review | | |
| **NAME OF DATABASE (interface):** Web of Science | | |
| **Concept** | **Line number** | **Search strategy** |
| Concept 1: | 1 | **((((((((((((((((((((((((((((((((((((((((((TS=(myoma)) OR TS=(myomatosis)) OR TS=(myomatous)) OR TS=(myomectomy)) OR TS=(myomectomie)) OR TS=(myomectomies)) OR TS=(myomatectomy)) OR TS=(myomatectomie)) OR TS=(myomatectomys)) OR TS=(myomatectomies)) OR TS=(myomectomys)) OR TS=(myomotomy)) OR TS=(myomotomie)) OR TS=(myomotomys)) OR TS=(myomotomies)) OR TS=(fibroleiomyoma)) OR TS=(fibroleiomyomas)) OR TS=(fibroma)) OR TS=(fibromas)) OR TS=(fibromyomas)) OR TS=(leiomyofibroma)) OR TS=(leiomyofibromas)) OR TS=(leiomyomas)) OR TS=(myomas)) OR TS=(fibro-myoma)) OR TS=(fibro-myomas)) OR TS=(fibroid)) OR TS=(fibroids)) OR TS=(fibromyoma)) OR TS=(leiomyoma)) OR TS=(leiomyomata)) OR TS=(leiomyomatas)) OR TS=(leiomyomatous)) OR TS=(leyomyoma)) OR TS=(leyomyomas)) OR TS=(leyomyofibroma)) OR TS=(leyomyofibromas)) OR TS=(leyomyomata)) OR TS=(leyomyomatas)) OR TS=(leyomyomatous)) OR TS=(fibroleyomyoma)) OR TS=(fibroleyomyomas))** |
| Concept 2: | 2 | **((((((((((((((((((((((((((((((((((((((((((TS=(delivery)) OR TS=(deliverys)) OR TS=(deliveries)) OR TS=(deliverie)) OR TS=(birth)) OR TS=(caesarean)) OR TS=(caesareans)) OR TS=(cesarean)) OR TS=(cesareans)) OR TS=(ceasarean)) OR TS=(ceasareans)) OR TS=(caesarian)) OR TS=(caesarians)) OR TS=(cesarian)) OR TS=(cesarians)) OR TS=(ceasarian)) OR TS=(ceasarians)) OR TS=(fetectomy)) OR TS=(fetectomie)) OR TS=(fetectomys)) OR TS=(fetectomies)) OR TS=(cesarotomy)) OR TS=(cesarotomie)) OR TS=(cesarotomies)) OR TS=(cesarotomys)) OR TS=(caesarotomy)) OR TS=(caesarotomie)) OR TS=(caesarotomies)) OR TS=(caesarotomys)) OR TS=(ceasarotomy)) OR TS=(ceasarotomie)) OR TS=(ceasarotomies)) OR TS=(ceasarotomys)) OR TS=(section)) OR TS=(sections)) OR TS=(sectio)) OR TS=(sectios)) OR TS=(labor)) OR TS=(labour)) OR TS=(parturition)) OR TS=(parturitions)) OR TS=(parturitional))** |
| Concept 3: | 3 | **((((((((((((((((((TS=(birth AND complications)) OR TS=(birth AND complication)) OR TS=(labor AND complication)) OR TS=(labour AND complication)) OR TS=(labor AND complications)) OR TS=(labour AND complications)) OR TS=(peri-partum AND complication)) OR TS=(peri-partum AND complications)) OR TS=(peripartum AND complication)) OR TS=(peripartum AND complications)) OR TS=(hysterorrhexis)) OR TS=(metrorrhexis)) OR TS=(ruptura uteri)) OR TS=(rupture AND uterus)) OR TS=(ruptured AND uterus)) OR TS=(rupture AND uterine)) OR TS=(ruptures AND uterus)) OR TS=(ruptures AND uterine))** |
| Filter / search block: | 4 | / |
| Combination of concepts | 5 | **((((((((((((((((((((((((((((((((((((((((((TS=(myoma)) OR TS=(myomatosis)) OR TS=(myomatous)) OR TS=(myomectomy)) OR TS=(myomectomie)) OR TS=(myomectomies)) OR TS=(myomatectomy)) OR TS=(myomatectomie)) OR TS=(myomatectomys)) OR TS=(myomatectomies)) OR TS=(myomectomys)) OR TS=(myomotomy)) OR TS=(myomotomie)) OR TS=(myomotomys)) OR TS=(myomotomies)) OR TS=(fibroleiomyoma)) OR TS=(fibroleiomyomas)) OR TS=(fibroma)) OR TS=(fibromas)) OR TS=(fibromyomas)) OR TS=(leiomyofibroma)) OR TS=(leiomyofibromas)) OR TS=(leiomyomas)) OR TS=(myomas)) OR TS=(fibro-myoma)) OR TS=(fibro-myomas)) OR TS=(fibroid)) OR TS=(fibroids)) OR TS=(fibromyoma)) OR TS=(leiomyoma)) OR TS=(leiomyomata)) OR TS=(leiomyomatas)) OR TS=(leiomyomatous)) OR TS=(leyomyoma)) OR TS=(leyomyomas)) OR TS=(leyomyofibroma)) OR TS=(leyomyofibromas)) OR TS=(leyomyomata)) OR TS=(leyomyomatas)) OR TS=(leyomyomatous)) OR TS=(fibroleyomyoma)) OR TS=(fibroleyomyomas)) AND ((((((((((((((((((((((((((((((((((((((((((TS=(delivery)) OR TS=(deliverys)) OR TS=(deliveries)) OR TS=(deliverie)) OR TS=(birth)) OR TS=(caesarean)) OR TS=(caesareans)) OR TS=(cesarean)) OR TS=(cesareans)) OR TS=(ceasarean)) OR TS=(ceasareans)) OR TS=(caesarian)) OR TS=(caesarians)) OR TS=(cesarian)) OR TS=(cesarians)) OR TS=(ceasarian)) OR TS=(ceasarians)) OR TS=(fetectomy)) OR TS=(fetectomie)) OR TS=(fetectomys)) OR TS=(fetectomies)) OR TS=(cesarotomy)) OR TS=(cesarotomie)) OR TS=(cesarotomies)) OR TS=(cesarotomys)) OR TS=(caesarotomy)) OR TS=(caesarotomie)) OR TS=(caesarotomies)) OR TS=(caesarotomys)) OR TS=(ceasarotomy)) OR TS=(ceasarotomie)) OR TS=(ceasarotomies)) OR TS=(ceasarotomys)) OR TS=(section)) OR TS=(sections)) OR TS=(sectio)) OR TS=(sectios)) OR TS=(labor)) OR TS=(labour)) OR TS=(parturition)) OR TS=(parturitions)) OR TS=(parturitional)) AND ((((((((((((((((((TS=(birth AND complications)) OR TS=(birth AND complication)) OR TS=(labor AND complication)) OR TS=(labour AND complication)) OR TS=(labor AND complications)) OR TS=(labour AND complications)) OR TS=(peri-partum AND complication)) OR TS=(peri-partum AND complications)) OR TS=(peripartum AND complication)) OR TS=(peripartum AND complications)) OR TS=(hysterorrhexis)) OR TS=(metrorrhexis)) OR TS=(ruptura uteri)) OR TS=(rupture AND uterus)) OR TS=(ruptured AND uterus)) OR TS=(rupture AND uterine)) OR TS=(ruptures AND uterus)) OR TS=(ruptures AND uterine))** |

| **Research question (e.g. PICO format):**  P: pregnant patients with a myomectomy in their medical history  I: labour  C: -  O: risk of uterine rupture | | |
| --- | --- | --- |
| **Type of review (e.g. systematic review, scoping review, narrative review, etc.):** Systematic review | | |
| **NAME OF DATABASE (interface):** The Cochrane Library | | |
| **Concept** | **Line number** | **Search strategy** |
| Concept 1: | 1 | 'myoma':ti,ab,kw OR 'myomatosis':ti,ab,kw OR 'myomatous':ti,ab,kw OR 'myomectomie':ti,ab,kw OR 'myomectomies':ti,ab,kw OR 'myomatectomy':ti,ab,kw OR 'myomatectomie':ti,ab,kw OR 'myomatectomys':ti,ab,kw OR 'myomatectomies':ti,ab,kw OR 'myomectomy':ti,ab,kw OR 'myomectomys':ti,ab,kw OR 'myomotomy':ti,ab,kw OR 'myomotomie':ti,ab,kw OR 'myomotomys':ti,ab,kw OR 'myomotomies':ti,ab,kw OR 'fibroleiomyoma':ti,ab,kw OR 'fibroleiomyomas':ti,ab,kw OR 'fibroma':ti,ab,kw OR 'fibromas':ti,ab,kw OR 'fibromyomas':ti,ab,kw OR 'leiomyofibroma':ti,ab,kw OR 'leiomyofibromas':ti,ab,kw OR 'leiomyomas':ti,ab,kw OR 'myomas':ti,ab,kw OR 'fibro-myoma':ti,ab,kw OR 'fibro-myomas':ti,ab,kw OR 'fibroid':ti,ab,kw OR 'fibroids':ti,ab,kw OR 'fibromyoma':ti,ab,kw OR 'leiomyoma':ti,ab,kw OR 'leiomyomata':ti,ab,kw OR 'leiomyomatas':ti,ab,kw OR 'leiomyomatous':ti,ab,kw OR 'leyomyoma':ti,ab,kw OR 'leyomyomas':ti,ab,kw OR 'leyomyofibroma':ti,ab,kw OR 'leyomyofibromas':ti,ab,kw OR 'leyomyomata':ti,ab,kw OR 'leyomyomatas':ti,ab,kw OR 'leyomyomatous':ti,ab,kw OR 'fibroleyomyoma':ti,ab,kw OR 'fibroleyomyomas':ti,ab,kw |
| Concept 2: | 2 | 'delivery':ti,ab,kw OR 'deliverys':ti,ab,kw OR 'deliveries':ti,ab,kw OR 'deliverie':ti,ab,kw OR 'birth':ti,ab,kw OR 'caesarean':ti,ab,kw OR 'caesareans':ti,ab,kw OR 'cesarean':ti,ab,kw OR 'cesareans':ti,ab,kw OR 'ceasarean':ti,ab,kw OR 'ceasareans':ti,ab,kw OR 'caesarian':ti,ab,kw OR 'caesarians':ti,ab,kw OR 'cesarian':ti,ab,kw OR 'cesarians':ti,ab,kw OR 'ceasarian':ti,ab,kw OR 'ceasarians':ti,ab,kw OR 'fetectomy':ti,ab,kw OR 'fetectomie':ti,ab,kw OR 'fetectomys':ti,ab,kw OR 'fetectomies':ti,ab,kw OR 'cesarotomy':ti,ab,kw OR 'cesarotomie':ti,ab,kw OR 'cesarotomies':ti,ab,kw OR 'cesarotomys':ti,ab,kw OR 'caesarotomy':ti,ab,kw OR 'caesarotomie':ti,ab,kw OR 'caesarotomies':ti,ab,kw OR 'caesarotomys':ti,ab,kw OR 'ceasarotomy':ti,ab,kw OR 'ceasarotomie':ti,ab,kw OR 'ceasarotomies':ti,ab,kw OR 'ceasarotomys':ti,ab,kw OR 'section':ti,ab,kw OR 'sections':ti,ab,kw OR 'sectio':ti,ab,kw OR 'sectios':ti,ab,kw OR 'labor':ti,ab,kw OR 'labour':ti,ab,kw OR 'parturition':ti,ab,kw OR 'parturitions':ti,ab,kw OR 'parturitional':ti,ab,kw |
| Concept 3: | 3 | ('birth':ti,ab,kw AND 'complications':ti,ab,kw) OR ('birth':ti,ab,kw AND 'complication':ti,ab,kw) OR ('labor':ti,ab,kw AND 'complication':ti,ab,kw) OR ('labour':ti,ab,kw AND 'complication':ti,ab,kw) OR ('labor':ti,ab,kw AND 'complications':ti,ab,kw) OR ('labour':ti,ab,kw AND 'complications':ti,ab,kw) OR ('peri-partum':ti,ab,kw AND 'complication':ti,ab,kw) OR ('peri-partum':ti,ab,kw AND 'complications':ti,ab,kw) OR ('peripartum':ti,ab,kw AND 'complication':ti,ab,kw) OR ('peripartum':ti,ab,kw AND 'complications':ti,ab,kw) OR 'hysterorrhexis':ti,ab,kw OR 'metrorrhexis':ti,ab,kw OR 'ruptura uteri':ti,ab,kw OR ('rupture':ti,ab,kw AND 'uterus':ti,ab,kw) OR ('ruptured':ti,ab,kw AND 'uterus':ti,ab,kw) OR ('uterine':ti,ab,kw AND 'rupture':ti,ab,kw) OR ('ruptures':ti,ab,kw AND 'uterus':ti,ab,kw) OR ('uterine':ti,ab,kw AND 'ruptures':ti,ab,kw) |
| Filter / search block: | 4 | / |
| Combination of concepts | 5 | 1 AND 2 AND 3  ('myoma':ti,ab,kw OR 'myomatosis':ti,ab,kw OR 'myomatous':ti,ab,kw OR 'myomectomie':ti,ab,kw OR 'myomectomies':ti,ab,kw OR 'myomatectomy':ti,ab,kw OR 'myomatectomie':ti,ab,kw OR 'myomatectomys':ti,ab,kw OR 'myomatectomies':ti,ab,kw OR 'myomectomy':ti,ab,kw OR 'myomectomys':ti,ab,kw OR 'myomotomy':ti,ab,kw OR 'myomotomie':ti,ab,kw OR 'myomotomys':ti,ab,kw OR 'myomotomies':ti,ab,kw OR 'fibroleiomyoma':ti,ab,kw OR 'fibroleiomyomas':ti,ab,kw OR 'fibroma':ti,ab,kw OR 'fibromas':ti,ab,kw OR 'fibromyomas':ti,ab,kw OR 'leiomyofibroma':ti,ab,kw OR 'leiomyofibromas':ti,ab,kw OR 'leiomyomas':ti,ab,kw OR 'myomas':ti,ab,kw OR 'fibro-myoma':ti,ab,kw OR 'fibro-myomas':ti,ab,kw OR 'fibroid':ti,ab,kw OR 'fibroids':ti,ab,kw OR 'fibromyoma':ti,ab,kw OR 'leiomyoma':ti,ab,kw OR 'leiomyomata':ti,ab,kw OR 'leiomyomatas':ti,ab,kw OR 'leiomyomatous':ti,ab,kw OR 'leyomyoma':ti,ab,kw OR 'leyomyomas':ti,ab,kw OR 'leyomyofibroma':ti,ab,kw OR 'leyomyofibromas':ti,ab,kw OR 'leyomyomata':ti,ab,kw OR 'leyomyomatas':ti,ab,kw OR 'leyomyomatous':ti,ab,kw OR 'fibroleyomyoma':ti,ab,kw OR 'fibroleyomyomas':ti,ab,kw) AND ('delivery':ti,ab,kw OR 'deliverys':ti,ab,kw OR 'deliveries':ti,ab,kw OR 'deliverie':ti,ab,kw OR 'birth':ti,ab,kw OR 'caesarean':ti,ab,kw OR 'caesareans':ti,ab,kw OR 'cesarean':ti,ab,kw OR 'cesareans':ti,ab,kw OR 'ceasarean':ti,ab,kw OR 'ceasareans':ti,ab,kw OR 'caesarian':ti,ab,kw OR 'caesarians':ti,ab,kw OR 'cesarian':ti,ab,kw OR 'cesarians':ti,ab,kw OR 'ceasarian':ti,ab,kw OR 'ceasarians':ti,ab,kw OR 'fetectomy':ti,ab,kw OR 'fetectomie':ti,ab,kw OR 'fetectomys':ti,ab,kw OR 'fetectomies':ti,ab,kw OR 'cesarotomy':ti,ab,kw OR 'cesarotomie':ti,ab,kw OR 'cesarotomies':ti,ab,kw OR 'cesarotomys':ti,ab,kw OR 'caesarotomy':ti,ab,kw OR 'caesarotomie':ti,ab,kw OR 'caesarotomies':ti,ab,kw OR 'caesarotomys':ti,ab,kw OR 'ceasarotomy':ti,ab,kw OR 'ceasarotomie':ti,ab,kw OR 'ceasarotomies':ti,ab,kw OR 'ceasarotomys':ti,ab,kw OR 'section':ti,ab,kw OR 'sections':ti,ab,kw OR 'sectio':ti,ab,kw OR 'sectios':ti,ab,kw OR 'labor':ti,ab,kw OR 'labour':ti,ab,kw OR 'parturition':ti,ab,kw OR 'parturitions':ti,ab,kw OR 'parturitional':ti,ab,kw) AND (('birth':ti,ab,kw AND 'complications':ti,ab,kw) OR ('birth':ti,ab,kw AND 'complication':ti,ab,kw) OR ('labor':ti,ab,kw AND 'complication':ti,ab,kw) OR ('labour':ti,ab,kw AND 'complication':ti,ab,kw) OR ('labor':ti,ab,kw AND 'complications':ti,ab,kw) OR ('labour':ti,ab,kw AND 'complications':ti,ab,kw) OR ('peri-partum':ti,ab,kw AND 'complication':ti,ab,kw) OR ('peri-partum':ti,ab,kw AND 'complications':ti,ab,kw) OR ('peripartum':ti,ab,kw AND 'complication':ti,ab,kw) OR ('peripartum':ti,ab,kw AND 'complications':ti,ab,kw) OR 'hysterorrhexis':ti,ab,kw OR 'metrorrhexis':ti,ab,kw OR 'ruptura uteri':ti,ab,kw OR ('rupture':ti,ab,kw AND 'uterus':ti,ab,kw) OR ('ruptured':ti,ab,kw AND 'uterus':ti,ab,kw) OR ('uterine':ti,ab,kw AND 'rupture':ti,ab,kw) OR ('ruptures':ti,ab,kw AND 'uterus':ti,ab,kw) OR ('uterine':ti,ab,kw AND 'ruptures':ti,ab,kw)) |
